# Mutated KRAS Is an Independent Negative Prognostic Factor for Survival in NSCLC Stage III Disease Treated with High-Dose Radiotherapy

**DOI:** 10.1155/2012/587424

**Published:** 2012-09-17

**Authors:** A. Hallqvist, F. Enlund, C. Andersson, H. Sjögren, A. Hussein, E. Holmberg, J. Nyman

**Affiliations:** ^1^Department of Oncology, Institute of Clinical Sciences, Sahlgrenska Academy at University of Gothenburg, 405 30 Gothenberg, Sweden; ^2^Department of Oncology, Sahlgrenska University Hospital, 413 45 Gothenburg, Sweden; ^3^Department of Pathology, Sahlgrenska University Hospital, 413 45 Gothenburg, Sweden; ^4^Department of Cytogenetics, Sahlgrenska University Hospital, 413 45 Gothenburg, Sweden

## Abstract

*Background*. The main attention regarding prognostic and predictive markers in NSCLC directs towards the EGFR-targeted pathway, where the most studied genetic alterations include EGFR mutations, EGFR copy number, and KRAS mutations. We wanted to explore the prognostic impact of mutated KRAS in the stage III setting treated with high-dose radiochemotherapy. *Methods*. Samples were obtained from patients participating in two prospective studies of locally advanced NSCLC receiving combined radiochemotherapy: the RAKET study, a randomized phase II study where patients were treated with induction chemotherapy (carboplatin/paclitaxel) followed by concurrent radiochemotherapy, and the Satellite trial, a phase II study with induction chemotherapy (cisplatin/docetaxel) followed by radiotherapy concurrent cetuximab. The samples were analysed regarding KRAS mutations, EGFR mutations, and EGFR FISH positivity. *Results*. Patients with mutated KRAS had a significantly inferior survival, which maintained its significance in a multivariate analysis when other possible prognostic factors were taken into account. The prevalence of KRAS mutations, EGFR mutations, and EGFR FISH positivity were 28.8%, 7.5%, and 19.7%, respectively. *Conclusion*. Mutated KRAS is an independent negative prognostic factor for survival in NSCLC stage III disease treated with combined radiochemotherapy. The prevalence of KRAS mutations and EGFR mutations are as expected in this Scandinavian population.

## 1. Introduction

The importance of finding prognostic and predictive markers is an ongoing challenge in oncology. The main attention regarding NSCLC directs towards the epidermal growth factor receptor- (EGFR) targeted pathway, where the most studied genetic alterations include EGFR mutations, EGFR copy number, and KRAS mutations. KRAS mutations have been shown to be associated with worse survival in resected patients [1, 2] and have also been shown to be a negative prognostic factor regarding adjuvant chemotherapy [3], whereas the significance in the stage III (locally advanced) or stage IV (metastatic) setting still is unclear.

In this study, we analysed tumour tissue samples regarding KRAS mutations, EGFR mutations, and EGFR positivity by FISH—high polysomy and amplification as defined by Cappuzzo et al. [4]—in patients with NSCLC stage III disease. The study population is represented by patients from two prospective trials who have received combined radiochemotherapy with curative intent: The RAKET study, a randomized three-armed phase II study where patients were treated with two cycles of induction chemotherapy (carboplatin/paclitaxel) followed by either hyperfractionated accelerated radiotherapy concurrent with a third cycle chemotherapy, conventional radiotherapy concurrent with daily paclitaxel or conventional radiotherapy concurrent with weekly paclitaxel [5]. Secondly the recently finished Satellite trial, a one-armed phase II study, where the treatment consisted of two cycles of induction chemotherapy (cisplatin/docetaxel) followed by radiotherapy concurrent with the EGFR-directed antibody cetuximab [6]. These two studies had the same inclusion criteria.

The aim was to explore the prognostic impact of KRAS mutations in the stage III setting where the patients have been treated with high-dose radiotherapy with concurrent chemotherapy/biotherapy and to survey the prevalence of KRAS and EGFR alterations in a Scandinavian population.

## 2. Patients and Methods

### 2.1. Patients

The patients involved in these analyses participated in the RAKET or Satellite study. The inclusion criteria were identical and consisted of histologically or cytologically proven nonresectable NSCLC stage IIIA/B (the 6th version of the TNM system), age > 18, FEV1 > 1.0 L or 40%, PS 0-1, and adequate bone marrow reserve. Main exclusion criteria were malignant pleural effusion, other malignancy treated within the last five years. Neither study excluded patients with weight loss. The RAKET study is a randomized national multicentre three-armed phase II study where patients were treated with two cycles of induction chemotherapy (carboplatin AUC 6/paclitaxel 200 mg/m^2^) followed by either (a) hyperfractionated accelerated radiotherapy 1.7 Gy BID to 64.6 Gy concurrent with a third-cycle chemotherapy, (b) radiotherapy with 2 Gy daily to 60 Gy concurrent with daily paclitaxel 12 mg/m^2^ or (c) radiotherapy with 2 Gy daily to 60 Gy concurrent with weekly paclitaxel 60 mg/m^2^. The study included 151 patients between 2002 and 2005. The Satellite trial is a one-armed multicentre phase II study where patients were treated with two cycles of induction chemotherapy (cisplatin 75 mg/m^2^/docetaxel 75 mg/m^2^) followed by radiotherapy, 2 Gy daily, to 68 Gy concurrent with weekly cetuximab (initial dose of 400 mg/m^2^ followed by 250 mg/m^2^). Seventy-five patients were recruited during 2006-2007. All patients gave their written informed consent and the studies were approved by the regional ethics board.

### 2.2. Tissue Specimen

Formalin-fixed paraffin-embedded tissues were collected from the diagnostic/pretherapeutic samples, and the analyses were performed at one site. The specimens were pathologically revised by a reference pathologist, and the section with highest percentage of tumour cells were estimated and marked, thereafter microdissected in preparation for the molecular analyses.

### 2.3. Molecular Analyses

The analysis of the KRAS- and EGFR mutations were performed by using TheraScreen KRAS Mutation Kit and TheraScreen EGFR29 Mutation Kit (DxS Diagnostics), detecting mutations in exon 2 and exon 18, 19, 21, respectively. EGFR copy number was analysed by using Vysis LSI EGFR SpectrumOrange/CEP7 SpectrumGreen Probe (Vysis Inc. Abbot laboratories), where the findings were classified according to Capuzzo et al. hence the term FISH positivity comprises high polysomy and amplification [4].

### 2.4. Statistics

The statistics for the main studies have been previously described [5, 6]. The OS survival analyses are done according to the Kaplan Meier method and possible univariate differences between groups are estimated with log-rank test. Univariate and multivariate Cox regression were done for several prognostic factors (i.e., KRAS mutation, performance status, gender, stage, and weight loss). In the multivariate Cox analyses the most significantly covariate was kept in the model and the other variables were entered into the model one by one and were maintained if they statistically improved the model on the 5% level.

## 3. Results

### 3.1. Main Results in the RAKET and Satellite Trials

The studies are as mentioned published previously but in short patients in the Satellite trial had a median survival of 17 months with a toxicity profile inferior to what usually is seen in concurrent chemoradiation. The RAKET trial had a median survival of 17.8 months, with all arms showing acceptable side effects, however there was no difference between the arms regarding local control or survival [5, 6].

### 3.2. Tissue Availability

All specimens from the patients in the Satellite trial were collected that previously had a biopsy performed, which were 34 out of 71 that is, 48%, the remaining patients were diagnosed on cytology. A group of the same size was chosen from the RAKET study using all tissue samples from the institution that randomized most of the patients. We hereby obtained 35 samples and as the number equals the sample number in the Satellite study, we then made a comparison between baseline characteristics in the whole study population and the subset with tissue specimen available (Table 1). The groups with tissue samples available show close to the same basal criteria as the whole population, with the exception of a slightly higher proportion of adenocarcinoma in the RAKET study group. 

The mean percentage of tumour cells in the samples, as assessed by the reference pathologist was 79% (range 10–100%).

### 3.3. KRAS Mutational Status (Tables 2 and 3)

**Figure 1:**
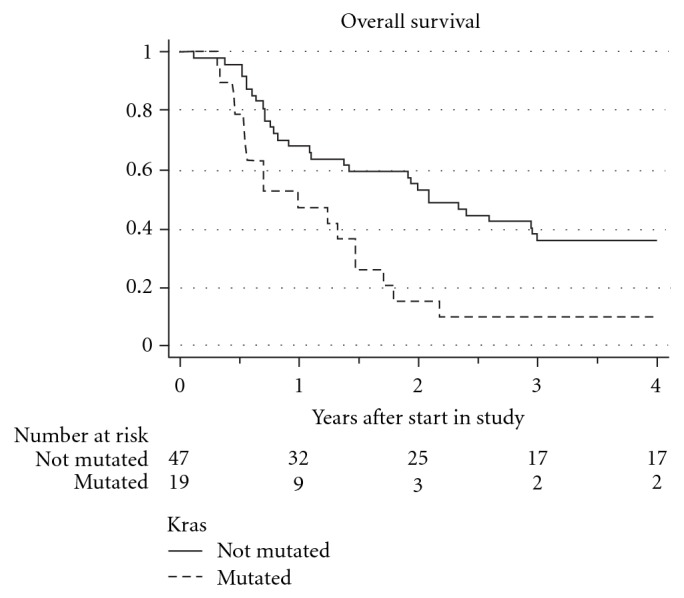
Survival depending on KRAS mutational status in the whole study population (HR 2.32, *P* = 0.006, 95% CI 1.27–4.26).

The prevalence of KRAS mutations was 28.8% (19 out of 66), where the majority was found in adenocarcinomas and a small proportion in squamous cell carcinoma (SCC, 10.5%). The group with mutated KRAS had a significantly inferior survival, which maintained its significance when introducing other possible prognostic factors into the cox regression model. They had previously shown to be of importance in the main study analyses, or are known to influence survival (i.e., performance status, gender, stage, and weight loss). The group difference is shown in Figure 1 with a hazard ratio of 2.32 (*P* = 0.006, 95% CI 1.27–4.26). The complete univariate and multivariate analyses are shown in Tables 4 and 5.

### 3.4. EGFR Mutational Status (Tables 2 and 3)

The prevalence of EGFR mutations were 7.5% (5 out of 67), one exon 19 deletion, one L858R (exon 21) mutation both in adenocarcinomas and three G719X mutations (exon 18) which were found in squamous cell carcinomas (SCC) and NSCLC NOS.

### 3.5. EGFR Gene Copy Number (Tables 2 and 3)

The prevalence of FISH positivity regarding EGFR was 19.7% (12 out of 61), where the prevalence in the Satellite and RAKET trial differed substantially 32.3% and 6.7% respectively. The histologies involved were in equal amounts SCC and adenocarcinoma with a lesser proportion NSCLC NOS. Four of the patients that were FISH positive had overlapping mutations, two with mutated KRAS, and two with mutated EGFR. 

## 4. Discussion

The knowledge about KRAS mutations and EGFR alterations, and their role in NSCLC, has expanded considerably in the last decade. KRAS mutations are more common in adenocarcinomas and smokers and are reported in 15–30% without any pronounced ethnical differences [7–10]. KRAS mutations have been shown to be associated with worse survival or higher frequency of relapses in resected patients [1, 2]. In a meta-analysis regarding all NSCLC stages, KRAS was a negative prognostic factor in terms of survival in univariate analysis but it was not analysed in the multivariate setting [11]. There is also data that KRAS is a negative prognostic factor for treatment with adjuvant chemotherapy [3, 8], whereas data regarding chemotherapy in the metastatic setting still is unclear. We have only found one study on the matter in stage III disease in which the patients received neoadjuvant radiochemotherapy followed by surgery where they found that KRAS was a negative prognostic factor regarding PFS in univariate analysis but a multivariate analysis was not performed [12]. In our study KRAS mutations were found in 28.8%, predominantly in adenocarcinomas, and this is in accordance with published data. When analysing KRAS mutation as a prognostic marker we found mutated KRAS to be a significant negative prognostic marker for survival which kept its significance in multivariate analysis when introducing other known possible prognostic factors, that is, performance status, gender, stage and weight loss, into the model. As far as we know this is the first time this is shown in a multivariate analysis in stage III disease treated with radiochemotherapy with a curative intent. The treatment with cetuximab in the Satellite trial might have interfered, as mutated KRAS is a biomarker of resistance to cetuximab in colorectal cancer [13], however in neither the FLEX- nor BMS099 trial where cetuximab was given combined with chemotherapy in stage IV disease, a correlation between KRAS and response to cetuximab in NSCLC [9] was observed.

We have also presented the frequencies of EGFR mutations and EGFR FISH positivity in this cohort, solely to give an idea of the prevalence in this Scandinavian population. The frequency of EGFR mutations differ for example, depending on histology, smoking status, and ethnicity. The highest figures are seen in Asian female never smokers with adenocarcinomas (>50%), whereas Caucasians have an overall mutational frequency of 7–10% which will rise to about 13–16% when considering adenocarcinomas [10, 14–16]. EGFR FISH positivity does not appear to have the same connection to histology or ethnicity but the prevalence varies substantially between 25 to >50% [7, 9, 14, 15, 17–20]. 

In this study, the EGFR mutation prevalence of 7% in a histologically unselected northern European Caucasian population is in accordance with previously published data, but two out of five patients had tumors originating from SCC. The pattern seen in the present study, with several exon 18 mutations in SCC differing from the most frequently reported pattern with the most common mutations being exon 19 deletions and exon 21 mutations, predominantly in adenocarcinomas. This is probably due to small sample size. Regarding FISH positivity the prevalence seems to be somewhat low (19.7%), with equal cases of adenocarcinomas and SCC. There is a substantial difference in the prevalence of FISH positive patients between the RAKET study and Satellite study where the prevalence is 6.7% and 32.3% respectively. We have no explanation for this difference, the samples were reanalysed with the same result.

In summary, we have shown that mutated KRAS is an independent negative prognostic factor for survival in NSCLC stage III disease treated with combined chemoradiotherapy or bioradiotherapy, and the prevalence of KRAS mutations and EGFR mutations are as expected in this northern European population. 

## Figures and Tables

**Table 1:** Baseline characteristics in the whole study population and the subset with tissue specimen available.

	RAKET		Satellite	
	Main study	Examined group	Main study	Examined group
*n* =	151	35	71	34
Median age	62 (43–78)	61 (49–78)	62 (42–81)	62 (43–76)
Gender F/M	48/52%	46/54%	51/49%	53/47%
PS 0/1	55/45%	40/60%	62/38%	65/35%
Stage IIIA/IIIB	34/66%	29/71%	37/62%	24/76%
Weight loss > 5%	17%	26%	37%	32%
FEV1	2.1 (0.8–4.5)	2.2 (0.9–4.3)	2.2 (1.1–3.7)	2.1 (1.1–3.7)
Histology:				
Adenocarcinoma	48%	63%	49%	44%
SCC	32%	26%	39%	44%
NSCLC NOS	20%	11%	12%	12%

**Table 2:** Prevalence, and relation to patient characteristics of EGFR mutations, KRAS mutations, and EGFR FISH positivity.

	EGFRmut	EGFR FISHpos	KRASmut
*n* =	5 = 7.5%	12 = 19.7%	19 = 28.8%
Inconclusive	2	8	3
Median age	62 (47–71)	62 (43–75)	62 (55–73)
Gender F/M	60/40%	67/33%	58/42%
PS 0/1	40/60%	50/50%	48/52%
Stage IIIA/IIIB	40/60%	17/83%	21/79%
Weight loss > 5%	20%	42%	32%
Histology			
Adenocarcinoma	40%	42%	68.5%
SCC	40%	42%	10.5%
NSCLC NOS	20%	16%	21%

**Table 3:** Prevalence depending on trial subset (RAKET or Satellite).

		RAKET			Satellite	
	EGFRmut	EGFR FISHpos	KRASmut	EGFRmut	EGFR FISHpos
*n* =	2∗	2∗∗	11	3^†^	10^††^	8
Inconclusive	2	5	3		3	
Median age	69, 71	69, 75	60 (55–73)	47, 54, 62	62 (43–68)	62 (60–67)
Gender F/M	F, F	F, M	55/45%	F, M, M	70/30%	62/38%
PS 0/1	1, 1	1, 1	45/55%	1, 0, 0	60/40%	50/50%
Stage IIIA/IIIB	IIIB, IIIB	IIIB, IIIB	18/82%	IIIA, 2IIIB	20/80%	25/75%
Weight loss > 5%	0	0, 1	27%	0, 0, 1	40%	37%
Histology:						
Adenocarcinoma	100%	100%	64%	0%	30%	75%
SCC	0%	0%	18%	67%	50%	0%
NSCLC NUS	0%	0%	18%	33%	20%	25%

∗ del 19 and L858R (exon 21).

∗∗one overlapping with EGFRmut L858R.

^†^G719X (exon 18).

^††^two overlapping with KRASmut and one with G719X.

**Table 4:** Univariate Cox-analyses.

Covariate	Number of patients	HR	95% confidence interval	*P* value
KRAS				
Not mutated	47	1		
Mutated	19	2.32	1.27–4.26	0.006
Gender				
Males	38	1		
Females	31	0.68	0.38–1.21	0.19
Age	69	0.99	0.96–1.03	0.67
PS				
0	36	1		
1	33	1.42	0.80–2.51	0.23
Stage				
IIIa	18	1		
IIIb	49	1.99	0.96–4.14	0.06
Weight loss				
<5%	49			
≥5%	20	1.79	0.98–3.27	0.06
Study				
RAKET	35	1		
Satellite	34	0.98	0.56–1.73	0.95

**Table 5:** Multivariate Cox regression. In the multivariate Cox regression no other covariate than mutated KRAS significantly improved the model.

Model number	Covariates	Number of patients	Hazard ratio (HR)	P	95% confidence interval
	Kras				
	Not mutated	45	1.00		
(1)	Mutated	19	2.32	0.010	1.21–4.13
	Stage				
	IIIa	16	1.00		
	IIIb	48	1.60	0.21	0.76–3.34

	Kras				
	Not mutated	47	1.00		
(2)	Mutated	19	2.32	0.005	1.30–4.44
	PS				
	0	34	1.00		
	1	32	1.42	0.23	0.80–2.51

	Kras				
	Not mutated	47	1.00		
(3)	Mutated	19	2.32	0.005	1.30–4.44
	Weight loss				
	Continues (kg)	66	1.05	0.30	0.95–1.15

	Kras				
	Not mutated	47	1.00		
(4)	Mutated	19	2.28	0.008	1.24–4.20
	Sex				
	Males	38			
	Females	31	0.63	0.132	0.35–1.15

	Kras				
	Not mutated	47	1.00		
(5)	Mutated	19	2.28	0.007	1.27–4.27
	Age				
	Continues (year)	66	0.99	0.524	0.95–1.03

	Kras				
	Not mutated	47	1.00		
(6)	Mutated	19	2.32	0.007	1.26–4.26
	Study				
	RAKET	32	1.00	0.77	0.52–1.63
	Satellite	34	0.92		
